# Identification of Thymus Specific and Developmentally Regulated Genes by an Improved Version of the mRNA Differential Display Technique

**DOI:** 10.1155/1999/58791

**Published:** 1999

**Authors:** Steve Pascolo, Debbie Tsoukatou, Clio Mamalaki

**Affiliations:** 1 lnterfakultäres Institut für Zellbiologie Abt. Immunologie Auf der Morgenstelle 15 Tübingen 72076 Germany; 2 lMBB FORTH P.O.Box 1527 Heraklion Crete 71110 Greece

**Keywords:** mRNA differential display technique, Thymus, development, mouse

## Abstract

During embryogenesis in mouse, the thymus is seeded by waves of hematopoietic stem cells that provide the first peripheral T lymphocytes after birth. It is known that embryo thymocytes and adult thymocytes have different phenotypic and functional features. The identification of genes expressed in the thymus only during embryogenesis would help to understand the molecular basis underlying these characteristics. We used the mRNA differential display technique to compare gene expression between thymus and kidney from embryo (171/2 days) and adult mice. This technique is the method of choice for comparing gene expression because it is able to display rapidly and simultaneously the mRNA complement from several different types of cells. The major drawback of the method is that it leads to the cloning of many false positives and therefore needs a high throughput method to screen for the truly differentially expressed cDNAs. We combined advantages from previously described methods in order to develop a new version of the mRNA differential display technique that is fast, cheap, and reliable. Instead of oligo dT priming, we used random hexameres for the reverse transcription of total RNA and 10-mer primers for the amplification of internal parts of the cDNAs. We obtained reproducible and clean patterns of discrete bands. We were able to easily identify DNAs differentially amplified between embryo and adult tissues (embryo specific; E 58.73), between thymus and kidney (thymus specific; Thy 52.54), or between embryo and adult thymus (embryo thymus specific; E Thy 58.73) cDNA fragments. After reamplification, cloning, and sequencing of these DNA fragments, it appeared that in most cases, one band corresponded to a single DNA sequence. On a northern blot, each of these candidate genes recognized a transcript that is differentially expressed as expected. Thus, we report an optimized, reproducible, and fast mRNA differential display method that overcomes the usual problems met with the originally described technique or its reported modifications.


*Developmental Immunology*, 1999, Vol. 7(1), pp. 1-7
Reprints available directly from the publisher
Photocopying permitted by license only

© 1999 OPA (Overseas Publishers Association)
N.V. Published by license under the
Harwood Academic Publishers imprint,
part of Gordon and Breach Publishing Group
Printed in Malaysia

### Identification of Thymus Specific and Developmentally Regulated Genes by an Improved Version of the mRNA Differential Display Technique

STEVE PASCOLO[a*], DEBBIE TSOUKATOU[b] and CLIO MAMALAKI[b]

[a]*Interfakultäres Institut für Zellbiologie, Abt. Immunologie Auf der Morgenstelle 15, 72076 Tübingen Germany and* [b]*IMBB, FORTH, P.O. Box 1527, 71110 Heraklion, Crete, Greece*

*(Received 31 July, 1998; Revised 18 September, 1998)*

During embryogenesis in mouse, the thymus is seeded by waves of hematopoietic stem cells that provide the first peripheral T lymphocytes after birth. It is known that embryo thymocytes and adult thymocytes have different phenotypic and functional features. The identification of genes expressed in the thymus only during embryogenesis would help to understand the molecular basis underlying these characteristics. We used the mRNA differential display technique to compare gene expression between thymus and kidney from embryo (171/2 days) and adult mice. This technique is the method of choice for comparing gene expression because it is able to display rapidly and simultaneously the mRNA complement from several different types of cells. The major drawback of the method is that it leads to the cloning of many false positives and therefore needs a high throughput method to screen for the truly differentially expressed cDNAs. We combined advantages from previously described methods in order to develop a new version of the mRNA differential display technique that is fast, cheap, and reliable. Instead of oligo dT priming, we used random hexameres for the reverse transcription of total RNA and 10-mer primers for the amplification of internal parts of the cDNAs. We obtained reproducible and clean patterns of discrete bands. We were able to easily identify DNAs differentially amplified between embryo and adult tissues (embryo specific; E 58.73), between thymus and kidney (thymus specific; Thy 52.54), or between embryo and adult thymus (embryo thymus specific; E Thy 58.73) cDNA fragments. After reamplification, cloning, and sequencing of these DNA fragments, it appeared that in most cases, one band corresponded to a single DNA sequence. On a northern blot, each of these candidate genes recognized a transcript that is differentially expressed as expected. Thus, we report an optimized, reproducible, and fast mRNA differential display method that overcomes the usual problems met with the originally described technique or its reported modifications.

*Keywords:* mRNA differential display technique, thymus, development, mouse

### INTRODUCTION

Programs of differential gene expression mediate cell differentiation that leads in higher eukaryotes to all specific biological processes, including development, organ functions, adaptation to the environment, or pathological disorders such as cell malignancy. In order to recognize and control these cell phenotypes,

* Corresponding author: Tel: 00 49 7071 29 80 997. Fax: 00 49 7071 29 5653. E-mail. Steve. PASCOLO@UNI-TUEBINGEN.DE.

it is necessary to characterize genes differentially expressed between cell types. Recently, a rapid method for identification of genes involved in cell differentiation has been developed. This technique named the mRNA differential display (Liang and Pardee, 1992, reviewed in Liang and Pardee, 1995) seemed to be the most promising one since it is the fastest method to investigate differential gene expression between two or more cell types. It utilizes reverse transcription of RNA and PCR amplification to produce a population of DNA fragments that can be separated according to size. The identification of DNA fragments specific for one cell type leads to the characterization of genes differentially expressed. In order to overcome some problems met with the first described mRNA differential display technique, closely related methods like RAP-PCR (Welsh et al., 1992)) or modifications of the original method have been reported (Sokolov and Prockop, 1994; Guimaraes et al., 1995; Graf et al., 1997; Fislage et al., 1997; Von Stein et al., 1997; Matz et al., 1997). Nevertheless, the pattern of PCR products is frequently not reproducible and reveals a high background ("smearing" problem). Moreover, the reamplification of a DNA product eluted from the acrylamide gel, leads to the cloning of many different sequences of related lengths that makes difficult the identification of the DNA product that is differentially expressed (Fengsheng et al., 1994; Poirier et al., 1997; Consalez et al., 1996; Smith et al., 1997). We combined the advantages of reported techniques to develop an improved version of mRNA differential display using random primers for the reverse transciption and 10-mer primers for PCR amplification as first described by Sokolov and Prockop (1994). This leads to a high reproducibility, solves background problems, and allows efficient reamplification of, in most cases, a unique PCR product corresponding to a differentially expressed gene. This method is low in cost (only one reverse transcription reaction is necessary for each RNA sample) and requires small amounts of total RNA (2 μg of total RNA to perform amplification with 40 combinations of primers). It can be applied to different organisms (plants, A. Aggelis and A. Kanellis, personnal communication) without any modification. It can also be used for the characterization of non poly adenylated RNA (like some viral RNA or prokaryotic mRNA). We utilized this technique to compare gene expression between embryo and adult mouse thymocytes. Embryo (171/2 days) thymocytes differ from adult thymocytes in TCR gene rearrangements (Chien et al., 1987), susceptibility to deletion (Finkel et al., 1992), terminal transferase expression (Bogues et al., 1992), and precursor origin (Jotereau et al., 1987). Genes involved in these differences are of interest in immunology and may help to understand the development of T lymphocytes and their mechanisms of activation or apoptosis. With this mRNA differential display technique, two combinations of 10-mer primers were enough to identify mRNAs that are stage and tissue specific. One of these RNAs, IGF2, is already known to be expressed exclusively in embryonic tissues (Christofori et al., 1994). In addition, we analyzed two new differentially amplified cDNA fragments. Both products correspond to genes expressed in the thymus but not in the kidney; one is not developmentally regulated, but the other is downregulated in the adult thymus. We report here the conditions used to obtain reproducible, low background patterns of PCR products as well as efficient and specific reamplification of the resulting DNA fragments.

### RESULTS

Duplicate experiments were carried out using independent stocks of RNA for both embryo and adult thymus. Figure 1 shows the pattern of bands obtained for two combinations of primers. Each combination of primers gave a different number of discrete bands up to more than 500 bases in length. As shown in Fig. 1, the DNA products are identical when similar cDNA matrixes are used for PCR amplification (compare the two lanes of embryo thymus or adult thymus). This proves that our protocol leads to reproducible results. Moreover, the amplification of cDNA from embryo or adult kidney resulted in the recovery of mainly identical DNA products that are similar to the DNA products obtained from thymus cDNA. Some exceptions appear to be kidney specific or thymus specific cDNA fragments. The size of each band could be precisely determined by running the PCR products together with a sequencing reaction from a known plasmid (not shown). From the dried acrylamide gel, we cut out three bands that are differentially expressed and potentially corresponding to three genes of interest: One band of 313 nt is amplified from embryo but not from adult tissues (E 58.73), one band of 197 nt is amplified from thymus but not from kidney RNA (Thy 52.54), and one band of 132 nt is amplified from embryo thymus but not from embryo kidney or adult thymus (E Thy 58.73). Each band was reamplified directly from 2 μl of the solution containing the acrylamide piece. Increasing the amount of this matrix DNA was not necessary and even detrimental to the amplification, probably because of an excess of salts or urea contained in the solution in which the gel slice was incubated. Under these conditions, the PCR reamplification resulted in more than 100 ng of the DNA of interest without any other products as visualized by running 40 μl of the PCR reaction on a 2% agarose gel. PCR products were then ligated in pCR II vectors (Invitrogen) and independent clones were sequenced. We found unique sequences corresponding to the bands E 58.73 (8 clones sequenced) and E Thy 58.73 (5 clones sequenced). The third band appeared to be a mixture of two PCR products of exactly the same size (Thy 52.54 α and β) with a dominance of the α sequence (9 clones out of 12 sequenced) over the β sequence (3 clones out of 12 sequenced). These four different DNA products were compared to DNA sequences available in databases.

Thy 52.54 α and E Thy 58.73 have no significant homology with any reported DNA sequence.

On the contrary E 58.73 is a part of the last exon of the insulinlike growth factor II (IGF2). The primers BS 73 and BS 58 have an identity with their targeted IGF2 sequence of 7 and 6 nucleotides, respectively. IGF2 is widely expressed in the developing mouse embryo, but its expression is progressively extinguished in virtually all tissues after birth (Christofori et al., 1994). Thus, the band E 58 73 that appears on the differential display gel as an embryo specific cDNA fragment is a single DNA product truly corresponding to a gene expressed only during embryogenesis both in thymus and kidneys.

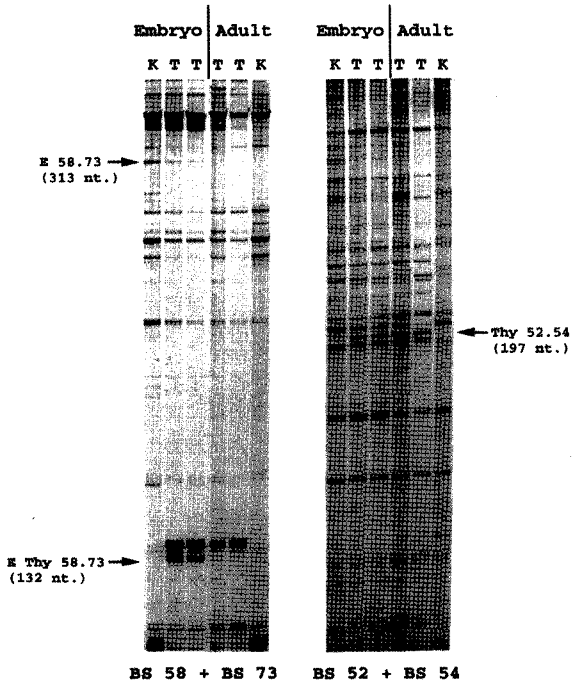

FIGURE 1 Size separation on a 5% sequencing gel of the RT PCR products obtained with the differential display procedure. cDNAs from embryo or adult kidney (K) and thymus (T) were amplified with the pair of primers BS 58 (5′CAGTGAGCGT3′) and BS 73 (5′AGCCTGTGTC3′) or BS 52 (5′CAAGCGAGGT3′) and BS 54 (5′AACGCGCAAC3′). For the thymus, duplicate experiments using two independant stocks of cDNA were performed. The arrows show the three bands that were cut out of the dried acrylamide gel for further analysis

The minor by-product obtained by reamplification of Thy 52.54 (the β sequence) is homologue with a FASTA score of 572 to the human RING 3 gene. This gene located in the MHC class II region has no known function in the immune system (Beck et al., (1996). When used as a probe on a northern blot, the Thy 52.54 β sequence recognizes a single mRNA transcript expressed both in kidney and thymus of embryo or adult mice (data not shown). So, the minor Thy 52.54 β product does not correspond to a gene differentially expressed; probably, it is a cDNA fragment weakly amplified by the primers BS 52 and BS 54 that is not visible on the differential display gel in the lanes corresponding to the kidney cDNAs. On the contrary, the major product obtained from the band Thy 52.54 (the α sequence) recognizes on a northern blot a single mRNA transcript of approximately 4 kb in total RNA from adult or embryo thymus but not in embryo kidney RNA (Fig. 2). Thus, the Thy 52.54 α product is corresponding to a mRNA differentially expressed between thymus and kidney in embryo as it appears to be on the differential display gel.

The fourth analyzed DNA sequence is a single DNA product corresponding to the cDNA fragment that appears to be amplified from the mouse embryo thymus cDNA. On a northern blot this sequence recognizes two RNA products; one major mRNA of approximately 4.5 kb and one minor of approximately 3 kb (Fig. 2). These two products are not detected in RNA from embryo kidneys and are weakly detected in RNA from adult thymus. Thus, as it appears on the differential display gel, this cDNA fragment corresponds to a gene expressed in the thymus but not in the kidney and upregulated during fetal development. Moreover, the long exposure time (1 week) that was required to visualize the mRNA product corresponding to E thy 58.73 in embryo thymus RNA, indicates that rare RNA species can be efficiently detected with our mRNA differential display technique.

### DISCUSSION

Trying to identify genes of the immune system that can be developmentally regulated, we used the mRNA differential display technology to compare the mRNA population of kidney and thymus from adult or 171/2 day mouse embryos. We describe a modified mRNA differential display technique that allowed us to characterize three genes differentially expressed with only two combinations of primers. We used random hexamers for the reverse transcription of the total RNA and 10-mer primers for the PCR amplification. This method has many advantages: (1) total RNA is used as starting material and only one reverse transcription reaction is required; (2) the PCR amplification with 10-mer primers gives a more reproducible and non-smearing pattern of bands compared with amplification with anchored oligo dT primers and can be used to study non polyadenylated RNA (like some viral nucleic acids or prokaryotic mRNA); (3) the reamplification of selected bands cut from acrylamide gels is easy and results in large amount of DNA; (4) in most of the cases, one band is a unique DNA product (although in other studies, we observed that reamplification of faint bands may lead to the cloning of more than two different DNA products); and (5) the 10-mer primers amplify internal parts of the cDNA that are more informative than the untranslated 3′ parts (as it was first described by Sokolov and Prockop, 1994) and can lead also to the identification of differentially spliced mRNA.

The reamplification of DNA products requires only one PCR reaction and leads to the cloning of a major species of DNA. Indeed, the amplification depends on the specificity of two primers contrarily to the originally described method in which only the 5′ 10-mer primer gave the specificity for the DNA to be amplified since the 3′ primer is an anchored oligo dT primer. The strategy we used for our method avoids totaly degenerated primers that are less efficient for reverse transcription compared with random hexamers (Kawasaki, 1990) and for PCR amplification compared with specific primers.

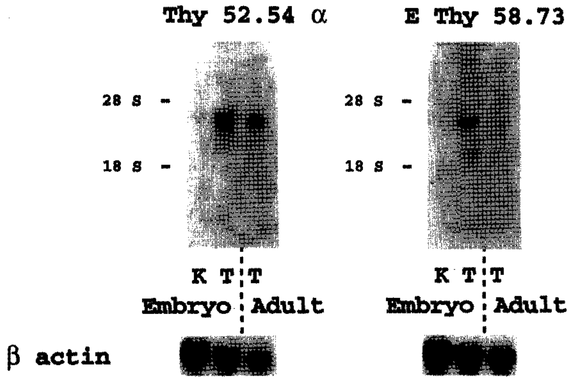

FIGURE 2 Northern blots hybridized with the cloned Thy 52.54 α (left panel) and E Thy 58.73 (right panel) DNA products. Total RNA from Kidney (K) or Thymus (T) from embryo or adult mice were size separated on an agarose/formaldehyde gel and blotted. The position of the ribosomic RNAs is indicated. Amounts of RNA were checked by hybridization of the same membranes with a β actin probe

We chose to analyze three bands differentially expressed and being (1) development specific (E 58.73); (2) organ specific (Thy 52.54), and (3) organ and development specific (E Thy 58.73). These three cDNA fragments were selected on the basis of an all-or-none criteria. Using this strategy, we attempted to identify genes turned on or off in a tissue or development specific manner. Nevertheless, users of our protocol reported that this method may be also quantitative since it allowed the identification of genes over- or underexpressed (A. Aggelis; data not shown). Moreover, the application of our improved mRNA differential display technique enabled us to identify a rare mRNA transcript that might be involved in T lymphocyte anergy (data not shown). These results indicate that the method is highly sensitive and not biased toward the detection of abundant mRNA species.

We could identify quickly the mRNA products corresponding to the three studied cDNA fragments and show that they are differentially expressed as expected. E 58.73 is corresponding to a part of the last exon of the IGF2 mRNA. This gene is expressed in all organs during embryogenesis and is downregulated after birth. The 5′ and 3′ primers are able to recognize the IGF2 transcript and they show an homology of 70 % and 60 % to their target sequence, respectively. Thy 52.54 α is corresponding to an unknown gene that is expressed in the thymus but not in the kidney of embryo mouse. Finally, E Thy 58.73 is a fragment of a gene expressed in the thymus of mouse embryo, not expressed in the kidney and downregulated in the adult thymus. We are currently analyzing the genes corresponding to E Thy 58.73 and Thy 52.54 α.

### MATERIAL AND METHODS

### Preparation of RNA

Thymuses and kidneys were removed from 171/2 days BALB/c embryos or from 4 to 6 week old adult Balb/c mice. Total RNA was prepared as described (Chomczynski and Sacchi, 1987). Briefly, small pieces of organs were teased in 2 ml of solution D (4 M guanidium thiocyanate, 25 mM sodium citrate, pH 7.0, 0.5% N-lauroylsarcosine, 100 mM β-mercapto-ethanol). Proteins and DNA were eliminated by acidic phenol extraction and the aqueous phase was again cleaned by neutral phenol/chloroform extraction. The RNA was recovered from the aqueous phase by isopropanol precipitation, resuspended in sterile water and quantified by O.D.

Thereafter, all work was carried out in conditions dedicated to avoid DNA contaminations arising from the presence of nucleic acids in all the tools and the air of standard molecular biology laboratories (Mc Pherson et al., 1992).

DNA-free RNA was obtained by DNAse treatment of 2 μg of total RNA for 1 hr at 37 °C in a final volume of 100 μl containing 40 mM Tris, pH 8, 10 mM NaCl, 6 mM $MgCl_2$, 40 u RNAsin (Promega), and 10 u DNase (Boehringer). After phenol/chloroform extraction and ethanol precipitation, the RNA was resuspended in 20 μl of sterile water.

### Reverse Transcription of RNA

One hundred pmol of random hexameres were added to the 20 μl of water containing 2 μg of DNA-free RNA. The samples were heated at 65°C for 10 min and cooled down to room temperature. The cDNA synthesis was carried out during 2 hr at 37°C in a final volume of 40 μl containing 500 μM dNTPs, 1 × RT buffer (Promega), 40 u RNasin (Promega), and 800 u M-MLV Reverse Transcriptase RNAse H minus (Promega).

In order to complete further the reverse transcription, the reactions were again heated at 65°C for 10 min and cooled down before the addition of 800 u extra of M-MLV RT RNAse H minus and incubation for 2 hr at 37°C before the addition of 1 μl of RNAse H (Promega) and 30 min incubation at 37°C.

### PCR Amplification

The reverse transcribed RNA was directly used for PCR amplification. We utilized the previously described 10-mer primers designed for PCR amplification of eukaryotic cDNAs (named BS 52, BS 54, BS 58, BS 73, etc; Sokolov and Prockop, 1994). 1 µl of cDNA (out of the 40 µl total volume of the reverse transcription reaction) was amplified in a final volume of 20 µl containing 10 mM Tris, pH 8, 25 mM KCl, 2 µM dNTPs, 0.5 µl dATP $^{35}S$ (10 mCi/ml; Amersham), 20 ng of each primer, and 1.5 mM $MgCl_2$. After the mix was overlayed with mineral oil, the PCR tubes were positioned in the thermocycler and heated up to 94°C. Then, 1 µl of Taq polymerase (diluted to 2.5 u/µl; Cetus) was added under the oil. The conditions of thermocycling were 94°C for 30 sec, 40°C for 2 min, 72°C for 30 sec; 40 cycles in a Perkin Elmer Cetus DNA thermal cycler. Then the samples were kept 10 min at 72°C before storage at 4°C. For the analysis of the PCR products, 5 µl of the reaction was denatured at 100°C in a mix containing 50% formamide, 5 mM EDTA, 0.01% xylene cyanol FF and 0.01% bromophenol blue and loaded on a 5% sequencing gel. At the end of the run, the acrylamide gel was dried and exposed to autoradiography film.

We observed differences in the pattern of bands coming from PCRs done in the same conditions but in different thermal cyclers or, in some cases, in different positions inside one thermal cycler (data not shown). Thus, particular attention should be dedicated to the selection of reagents and tools used to prepare the DNA-free RNA, the cDNA, and the PCR reactions.

### Recovery and Reamplification of cDNA Fragments

A differentially amplified cDNA was identified and recovered by precise cutting of a gel slice inside the band. This piece of dried acrylamide gel was incubated overnight at room temperature in 100 µl of water. Reamplification was achieved directly by using 2 µl out of this solution in a final volume of 50 µl containing PCR buffer (Cetus), 200 µM dNTPs, 2.5 mM $MgCl_2$, and 20 ng of each primer. The mix was overlayed with mineral oil. The PCR tubes positioned in the thermocycler were heated up to 94°C prior to the addition of 1 µl of Taq polymerase (diluted to 2.5 u/µl; Cetus) under the oil. The conditions of thermocycling were 94°C for 30 sec, 40°C for 2 min, 72°C for 30 sec; 40 cycles. Then the samples were kept for 10 min at 72° C before storage at 4 °C. Forty µl of each PCR reaction were loaded on a 2% agarose gel to check the efficiency and specificity of the reamplification. Then, 2 to 4 µl of PCR reaction were used for ligation in a pCR II vector (Invitrogen). The inserts were sequenced, and used as probes on northern blots.

***Acknowledgements***

We thank Annette Nassuth and Jutta Moosbauer for critical reading of the manuscript. This project was supported by Human Capital and Mobility CHRX-CT94–0584 grant. S.P. is a TMR Marie Curie Fellow contract #ERBFMBICT96- 1857.

***References***

Liang, P., and Pardee, A. (1992) Differential display of eukaryotic messenger RNA by means of the polymerase chain reaction. *Science*, **257**, 967–971.

Liang, P., and Pardee, A. (1995) Recent advances in differential display. *Curr. Opin. Immunol.*, **7**, 274–280.

Welsh, J., Chada, K., Dalal, S., Cheng, R., Ralph, D., and McClelland, M. (1992) Arbitrarily primed PCR fingerprinting of RNA. *Nucleic Acids Res.*, **20**, 4965–4970.

Sokolov, B., and Prockop, D. (1994) A rapid and simple PCR based method for isolation of cDNAs from differentially expressed genes. Nucleic Acids Res., 22, 4009–4015.

Guimaraes, J., Lee, F., Zlotnik, A., and McClanahan, T. (1995) Differential display by PCR: Novel findings and applications. *Nucleic Acids Res.*, **23**, 1832–1833.

Graf, D., Fisher, A., and Merkenschlager, M. (1997) Rational primer design greatly improves differential display-PCR (DD-PCR). *Nucleic Acids Res.*, **25**, 2239–2240.

Fislage, R., Berceanu, M., Humboldt, Y., Wendt, M., and Oberender, H. (1997) Primer design for prokaryotic differential display RT-PCR. *Nucleic Acids Res.*, **25**, 1830–1835.

Von Stein, O., Thies, W. G. and Hofmann, M. (1997) A high throughput screening for rarely transcribed differentially expressed genes. *Nucleic Acids Res.*, **25**, 2598–2602.

Matz, M., Usman, N., Shagin, D., Bogdanova, E., and Lukyanov, S. (1997) Ordered differential display : a simple method for systematic comparison of gene expression profiles. *Nucleic Acids Res.*, **25**, 2541–2542.

Fengsheng, L., Barnathan, E., and Kariko, K. (1994) Rapid method for screening and cloning cDNAs generated in differential mRNA display: application of Northern blot for affinity capturing of cDNAs. *Nucleic Acids Res.*, **22**, 1764–1765.

Poirier, G., Pyati, J., Wan, J., and Erlander, M. (1997) Screening differentially expressed cDNA clones obtained by differential display using amplified RNA. *Nucleic Acids Res.*, **25**, 913–914.

Consalez, G., Corradi, A., Ciarmatori, S., Bossolasco, M., Malgaretti, N., and Stayton, C. (1996) A new method to screen clones from differential display experiments prior to RNA studies. *TIG*, **12**, 455–456.

Smith, N., Li, A., Aldersley, M., High, A., Markham, A., and Robinson, P. (1997) Rapid determination of the complexity of cDNA bands extracted from DDRT-PCR polyacrylamide gels. *Nucleic Acids Res.*, **25**, 3552–3554.

Chien, Y., Iwashima, M., Kaplan, K., and Davis, E., and Davis, M. (1987) A new T-cell receptor gene located within the alpha locus and expressed early in T-cell differentiation. *Nature,* **327**, 677–682.

Finkel, T., Kappler, J., and Marrack, P. (1992) Immature thymocytes are protected from deletion early in ontogeny. Proc. *Natl. Acad. Sci. USA,* **89**, 3372–3374.

Bogues, M., Gilfillan, S., Benoist, C., and Mathis, D. (1992) Regulation of N-region diversity in antigen receptors through thymocyte differentiation and thymus ontogeny. *Proc. Natl. Acad.* Sci. USA, **89**, 11011–11015.

Jotereau, F., Heuze, F., Salomon-Vie, V., and Gascan, H. (1987) Cell kinetics in the fetal mouse thymus precursor cell input, proliferation and emigration. *J. Immunol.*, 138, 1026–1030.

Christofori, G., Naik, P., and Hanahan, D. (1994). A 2d signal supplied by insulin like Growth Factor II in oncogene-induced tumorigenesis. *Nature*, **369**, 414–416.

Chomczynski, P., and Sacchi, N. (1987) Single-step method of RNA isolation by acid guanidium thiocyanate-phenol chloroform extraction. *Anal. Biochem.*, **162**, 156–159.

Mc Pherson, M., Quirke, P., and Taylor, G. (1992). PCR, a practical approach. (OIRL Press).

Beck S., Belich, M., Gruneberg, U., Jackson, A., Kelly, A., Sanderson, P., Trowsdale, J., and Van Ham, M. (1996). Organisation and functions of class II genes and molecules. *DNA Seq.*, **7**, 21–23.

Kawasaki, E. (1990). Amplification of RNA. In PCR Protocols : A Guide to Methods and Applications, Innis, M. A., Gelfand, D. H., Sninsky, J. J., White, T. T. Eds (Orlando, FL: Academic Press).

